# Management of Unilateral Masseter Hypertrophy and Hypertrophic Scar—A Case Report

**DOI:** 10.1155/2012/521427

**Published:** 2012-07-10

**Authors:** Naresh Shetty, Rajanikanth K. Malaviya, M. K. Gupta

**Affiliations:** ^1^Department of Oral & Maxillofacial Surgery, Faculty of Dentistry, Melaka Manipal Medical College, 75150 Melaka, Malaysia; ^2^Department of Oral & Maxillofacial Surgery, Sharad Pawar Dental College, Wardha 442005, India; ^3^Department of Oral & Maxillofacial Surgery, Peoples Dental Academy, Bhopal 462010, India

## Abstract

Masseter muscle hypertrophy is a rare condition of idiopathic cause. It clinically presents as an enlargement of one or both masseter muscles. Most patients complain of facial asymmetry; however, symptoms such as trismus, protrusion, and bruxism may also occur. Several treatment options reported for masseter hypertrophy are present, which range from simple pharmacotherapy to more invasive surgical reduction. Keloid scar with unilateral masseter hypertrophy is a rarely seen in clinical practice. This paper reports a case of unilateral masseter hypertrophy with keloid scar in the angle of the mandible for which surgical treatment was rendered to the patient by using a single approach.

## 1. Introduction

Masseter hypertrophy is usually an asymptomatic enlargement of one or both masseter muscles. In majority of the cases, the etiology is idiopathic. The highest incidence for this condition is in the second and third decades of life, with no gender predilection. A congenital variety also exists, but acquired masseter hypertrophy is more common. Unilateral occurrence can be seen when patients chew or clench primarily on one side. Muscle function may also be impaired, thus causing conditions such as trismus, protrusion, and bruxism. Numerous factors such as malocclusion, bruxism, clenching, or temporomandibular joint disorders, have been cited. The accurate diagnosis is more difficult in unilateral cases. A hypertrophied masseter will alter facial lines, cause generating discomfort, and negative cosmetic impacts in many patients. Masseter hypertrophy leads to the prominent mandibular angle which is aesthetically unacceptable to the patient. The differential diagnosis includes parotid tumor, lipoma, benign or malignant muscle tumors, and vascular tumors.

## 2. Case Report

**Figure 1:**
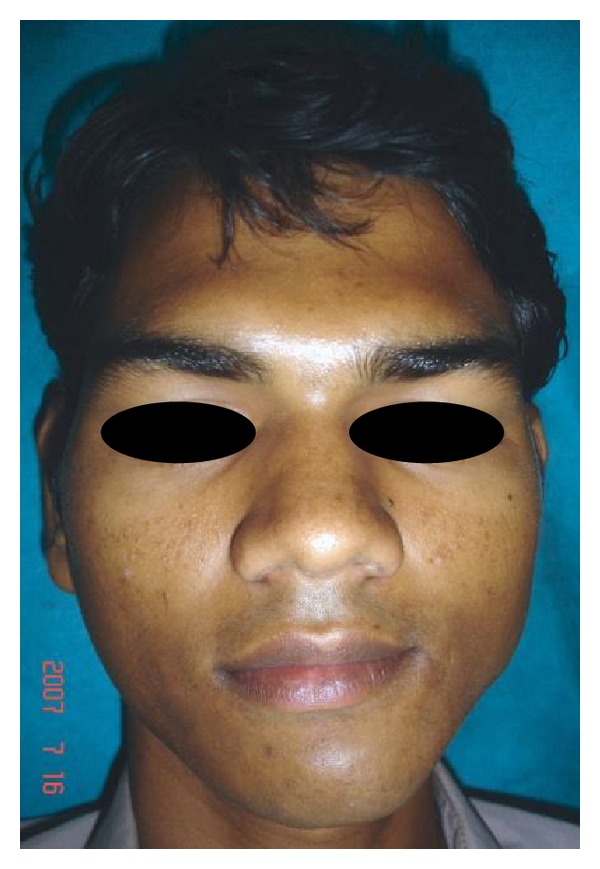
Pre-op frontal view.

**Figure 2:**
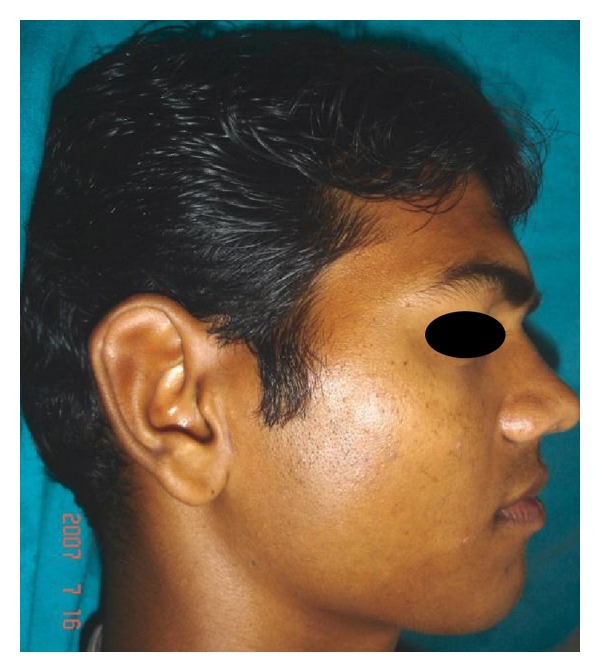
Pre-op right lateral view.

**Figure 3:**
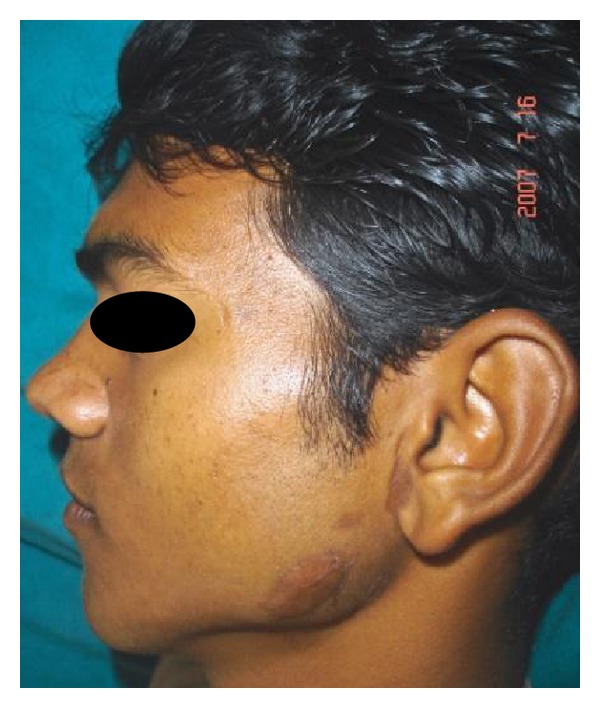
Pre-op left lateral view.

**Figure 4:**
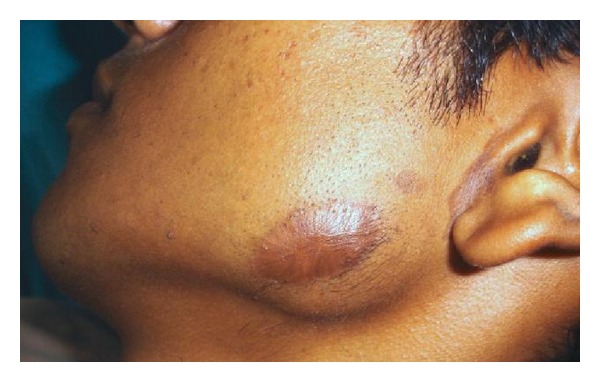
Pre-op left close-up view.

**Figure 5:**
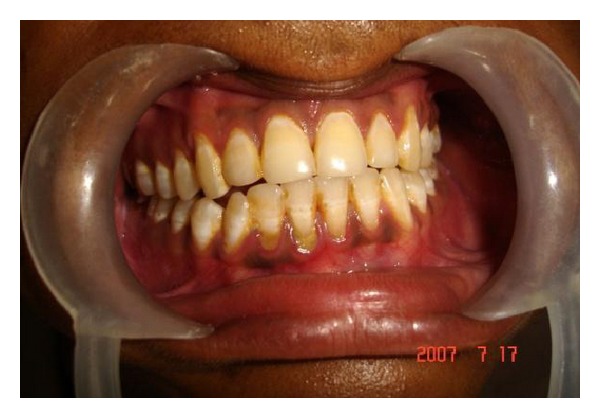
Pre-op occlusion.

**Figure 6:**
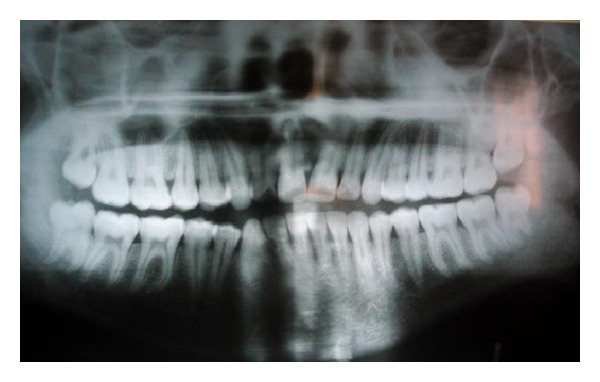
Pre-op OPG.

**Figure 7:**
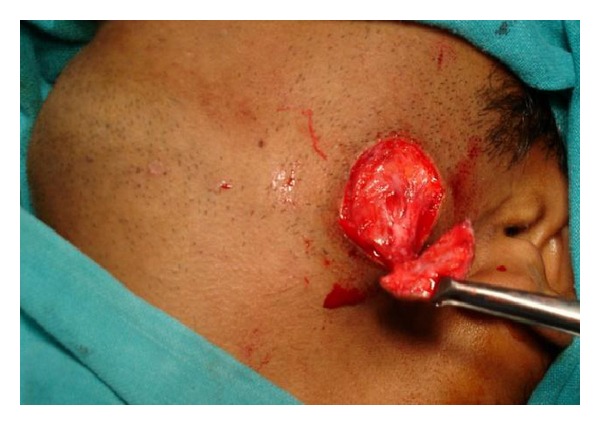
Intraoperative view.

**Figure 8:**
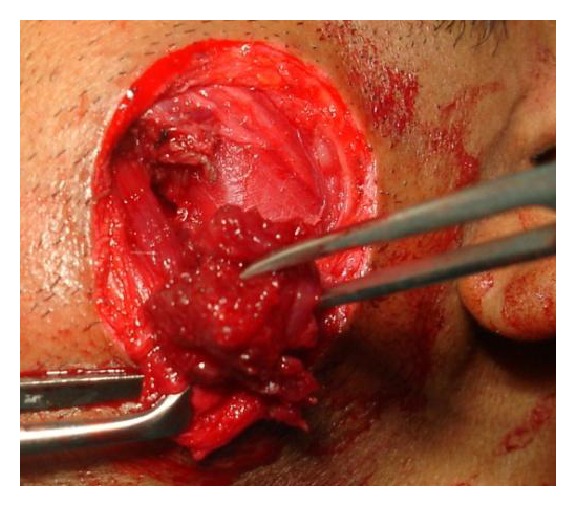
Hypertrophic muscle removed.

**Figure 9:**
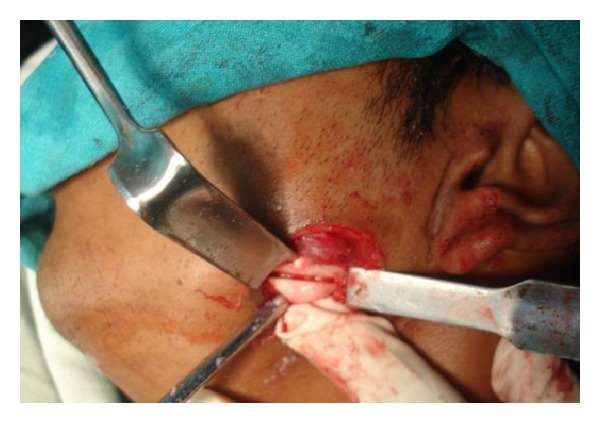
Bony excess resected at angle.

**Figure 10:**
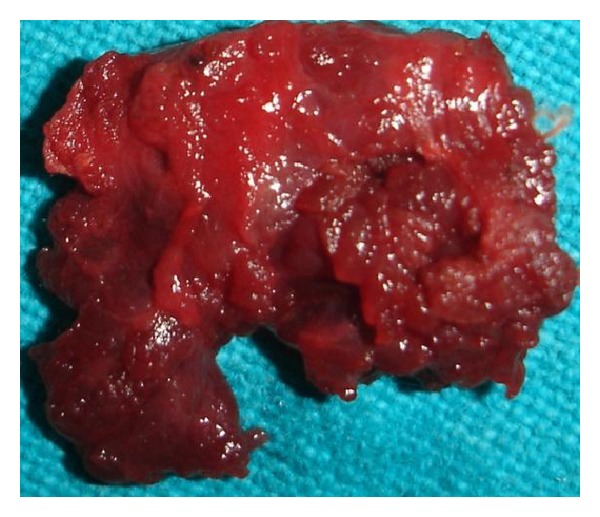
Resected masseter muscle.

**Figure 11:**
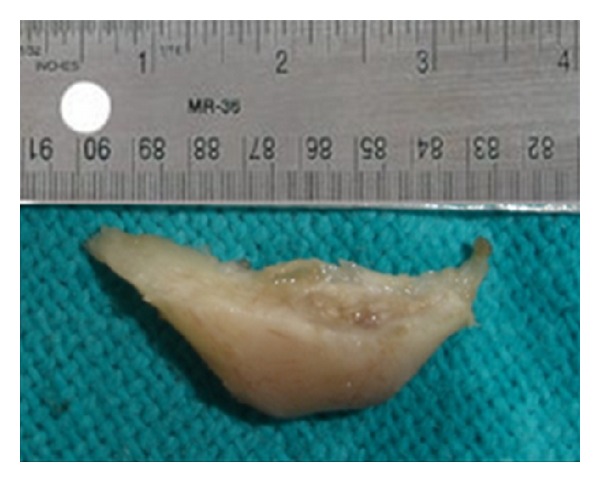
Resected angle.

**Figure 12:**
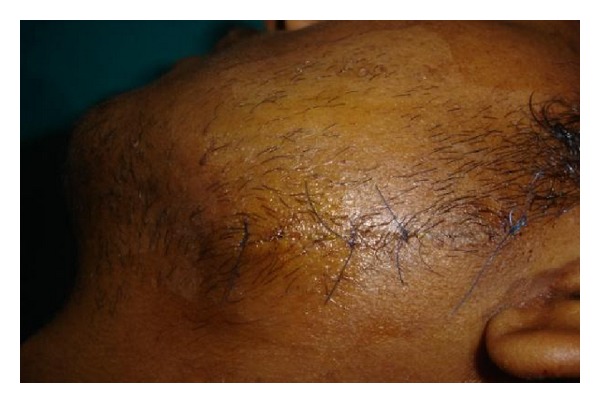
Sutures placed.

A 28-year-old male patient reported to the department of oral and maxillofacial surgery at people's dental academy complaining of asymmetry of the face and scar in the mandibular angle region on the left side since 3 years. The patient gave a history that he had tripped and fallen down 3 years back and he had a laceration on the left side of the face that developed in to a scar. The patient's chief complaint was left side facial growth without pain. The patient had no history of systemic diseases. Extraoral examination showed an obvious unilateral swelling centered over the mandibular angle. Palpation indicated that the swollen tissue was normal in tone and nontender. Mandibular movements were in the normal range. When the patient was asked to clench, the swelling became more prominent and firm. The patient said that he uses the left side of the jaw more while chewing food. There was no history of temporomandibular joint clicking, and no family history of masseter hypertrophy. Physical examination revealed that the patient had unilateral masseter muscle bulging, with a prominent mandibular angle at the lower border. Intraoral examination revealed distoangularly impacted 38 and 48. OPG showed a prominent mandibular angle. Data from clinical and radiographic examination led to the diagnosis of unilateral masseter muscle hypertrophy (Figures 1, 2, 3, 4, 5, and 6). Nonsurgical options such as botox therapy and the advantages and disadvantages of both surgery and botox treatment were discussed with the patient. The patient opted for surgical option as he wanted to get rid of the scar immediately and we told him that we can do the correction of masseter hypertrophy and scar revision through one incisional approach only. A combined reduction of the mandibular angle and shaving of the masseter muscle was planned. The surgery was done under general anesthesia with nasotracheal intubation. Xylocaine 2% with adrenalin was infiltrated in the angle of the mandible. An elliptical incision was placed around the hypertrophic scar and the scar was removed (Figure 7). The marginal mandibular nerve was identified and protected. Debulking of the masseter muscle was performed as the patient was very worried about the asymmetry of the face (Figure 8). The muscle was incised approximately 5 mm above the mandibular basilar. The entire ascending portion of the masseter muscle was detached, and a vertical internal muscle band equivalent to two third of the thickness of the muscle was resected. After the muscle was resected, the remaining external third was sutured to its site of origin onto the muscle stump inserted in the mandibular basilar. The bony deformity was trimmed and removed in the angle of the mandible with surgical bur (Figure 9). Sharp margins were trimmed with a bone file. The shaved masseter muscle and the resected excess mandibular angle was sent to oral pathology department in 10% formalin (Figures 10 and 11). Primary closure was done with 5-0 prolene suture (Figure 12). After 1 week, the prolene sutures were removed and the wound healed uneventfully.

## 3. Discussion

The masseter muscle is essential for adequate mastication and is located laterally to the mandibular ramus and thus plays an important role in facial esthetics. Diagnosis of masseter hypertrophy can be achieved from clinical examination, history, panoramic X-ray, and muscle palpation. The best diagnostic test is to palpate the masseter muscle with fingers, while the patient clenches his/her teeth so the muscle is more prominent during contraction. With the muscle is relaxed and the patient's mouth is slightly open, extraoral palpation with both hands will pinpoint the intramuscular location of the hypertrophy. Upon relaxation, the jaw angle may reveal irregularities that on the X-ray image may appear to be a bone increase. Idiopathic masseter muscle hypertrophy was first described by Legg in 1880, reporting on the case of a 10-year-old girl with concurrent idiopathic temporalis muscle hypertrophy [1]. According to Teixeira et al., there are two types of masseter muscle hypertrophy: congenital or familial and acquired due to functional hypertrophy [2]. There are various treatment modalities for the management of masseter hypertrophy. This can be categorized into nonsurgical and surgical. Management of the idiopathic masseter hypertrophy is based on psychological counseling, use of mouth guards, -muscle relaxant, and anxiolytic drugs, analgesics, physical therapy, dental restorations, and occlusal adjustments to correct premature contacts. A good result can be achieved in the patients with mild hypertrophy but there is no reliable report on the literature on the success rates of isolated clinical therapy. Injection of botulinum toxin type A into the masseter muscle is generally considered a less invasive modality and has been advocated for cosmetic sculpting of the lower face. Injection of botulinum toxin type A into the masseter muscle was first introduced by Smyth, Moore, and Wood in 1994 and considered a less invasive modality for the treatment of muscle hypertrophy [3]. Local injection of very small doses of the toxin into a muscle produces local paralysis and therefore, individual muscles can be selectively weakened and atrophy of the muscle occurs. Botulinum toxin type A is a powerful neurotoxin which is produced by the anaerobic organism clostridium botulinum and when injected into a muscle causes interference with the neurotransmitter mechanism producing selective paralysis and subsequent atrophy of the muscle. Perhaps the biggest disadvantage of botulinum toxin therapy is that the treatment effect wears away and reverts to the original condition in 6 months [4]. The traditional method of treatment for masseter hypertrophy is the surgical partial excision of masseter muscle under general anesthesia. The surgical treatment is based on intra- and extraoral approaches. Both techniques revolve around the removal of excessive muscle fibers from the inner third of the masseter vertical muscle fibers. Reduction osteoplasty may be performed in cases of bony hyperplasia of the mandibular angle [5]. The remaining external bundle of the masseter should be attached to the mandibular periosteum to allow for adequate functional recovery. The choice between intra- and extraoral approaches is not related to cosmetic or functional outcomes or to the risk of introducing vascular and nerve injury, but to the skill and experience of the surgeon in performing surgery using either of the approaches. In the beginning, the extraoral approach was widely indicated, because it offered better visualization. The procedure is carried out through a submandibular incision, Risdon. Unlike surgical excision of muscle tissue that reduces the actual number of muscle cells, botulinum toxin type A only reduces muscle volume temporarily. Surgical treatment was proposed for the first time by Gurney in 1947 [6]. The procedure consists of a sub-mandibular incision and the removal of three fourth to two third of all muscle tissue available from the muscle upper aponeurosis to the lower mandibular border. Removal of the masseter muscle insertion by means of a triangular incision was done by Martensson in a patient with history of bruxism and unilateral masseter muscle hypertrophy [7]. Beckers in 1977 surgically treated 17 patients using the intraoral approach in which internal muscle band was removed from the hypertrophied masseter. An internal muscle band was removed from the hypertrophied masseter from the upper insertion in the zygomatic arc to the lower insertion in the mandibular angle, thus avoiding the production of a visible scar on the patient's face and reducing the possibility of injuring branches of the facial nerve [8]. Another surgical technique is, in which the bony protuberance is removed from the mandibular angle without removing any parts of the masseter muscle [9]. Complications from surgical excision of masseter include hematoma formation, facial nerve paralysis, infection, mouth opening limitation and sequelae from general anesthesia [10]. Nonsurgical approaches such as botox therapy have both advantages and disadvantages to surgical approaches [11, 12].

## 4. Conclusion

The masseter hypertrophy was removed along with hypertrophic scar. With a single surgery treatment of masseteric hypertrophy and hypertrophic scar have been carried out which is probably the first time and long-term followup is required following the surgery.
